# Management and treatment of chronic kidney disease in the Danish Lolland-Falster Health Study: focus on renoprotection and cardiovascular disease prevention

**DOI:** 10.1093/ckj/sfaf242

**Published:** 2025-08-01

**Authors:** Ebba Mannheimer, Morten Buus Jørgensen, Kristine Hommel, Anne-Lise Kamper, Randi Jepsen, Bo Feldt-Rasmussen, Mads Hornum

**Affiliations:** Department of Nephrology and Endocrinology, Rigshospitalet, Copenhagen, Denmark; Department of Nephrology and Endocrinology, Rigshospitalet, Copenhagen, Denmark; Department of Medicine, Holbæk Hospital, Holbæk, Denmark; Department of Clinical Medicine, Faculty of Health and Medical Sciences, University of Copenhagen, Copenhagen, Denmark; Department of Nephrology and Endocrinology, Rigshospitalet, Copenhagen, Denmark; Lolland-Falster Health Study, Centre for Health Research, Zealand University Hospital, Nykøbing F., Denmark; Department of Nephrology and Endocrinology, Rigshospitalet, Copenhagen, Denmark; Department of Clinical Medicine, Faculty of Health and Medical Sciences, University of Copenhagen, Copenhagen, Denmark; Department of Nephrology and Endocrinology, Rigshospitalet, Copenhagen, Denmark; Department of Clinical Medicine, Faculty of Health and Medical Sciences, University of Copenhagen, Copenhagen, Denmark

**Keywords:** blood pressure, cardiovascular disease, chronic kidney disease, epidemiology, renoprotection

## Abstract

**Background:**

In the Danish population-based Lolland-Falster Health Study (LOFUS), we recently identified a chronic kidney disease (CKD) prevalence of 18%. Importantly, overall disease recognition was only 7.1%, and awareness was as low as 4.4%. This reveals a significant gap in identifying CKD, consequently delaying initiation of guideline-directed renoprotective treatments, cardiovascular disease prevention, and referrals to specialized nephrology care.

**Methods:**

Cross-sectional study including adult participants with CKD identified in LOFUS. Data were obtained from biochemical analyses, clinical examinations, and questionnaires. Redeemed prescriptions and nephrology referrals were assessed using national medical registers. Blood pressure control, treatment with renin–angiotensin–system inhibitors and statins, as well as lifestyle factors were examined, and their association with CKD stage analyzed.

**Results:**

Among 2881 individuals with CKD, 57.6% were women, median age was 67.8 years, 71.3% were in CKD stages 1–2 and 21% had cardiovascular disease. Less than half of individuals had blood pressure control (47.5%). Treatment with renin–angiotensin–system inhibitors and statins, when indicated, were 72.8% and 32.2%, respectively, and more frequent in individuals with diabetes. In multivariable analyses, the odds ratios for blood pressure control (1.68; 95% CI,1.12–2.52), treatment with renin–angiotensin–system inhibitors (7.91; 95% CI,2.14–29.18), and statins (1.77; 95% CI,1.06–2.96) were significantly higher in stages 3b–5 compared to stage 1. Less than one-third had a BMI <25 kg/m^2^ and >80% self-reported non-smoking. Of those meeting nephrology referral criteria (*n *= 99), one-third had been referred.

**Conclusion:**

Our findings highlight gaps between guideline-recommended CKD management and practice, particularly in early stages and in non-diabetic individuals, emphasizing the need for early detection and improved guideline adherence.

## GRAPHICAL ABSTRACT

**GRAPHICAL ABSTRACT:**
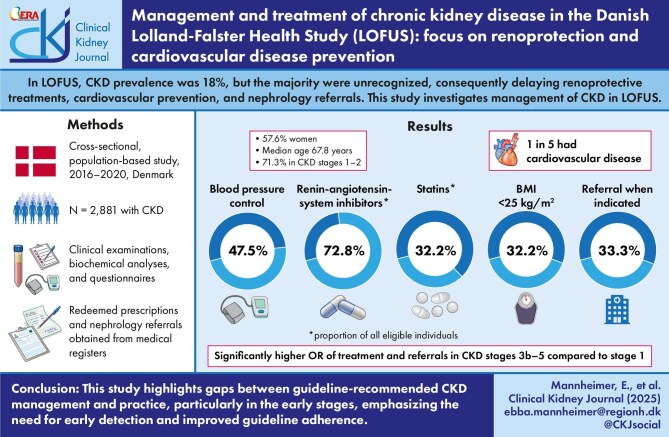

KEY LEARNING POINTS **What was known:**

- Chronic kidney disease (CKD) is a major cause of mortality, mainly due to increased cardiovascular risk.
- In the Danish Lolland-Falster Health Study, we identified a CKD prevalence of 18%, however, disease recognition was only 7%.
- This highlights a gap in CKD identification, delaying renoprotective treatments, cardiovascular disease prevention, and nephrology referrals.

**This study adds:**

- Although RAS inhibitor use was high when indicated, blood pressure control remained below 50% but was significantly more likely in stages 3–5, compared to stage 1.
- Cardiovascular disease was common, and fewer than one-third received statins when indicated or had BMI <25 kg/m^2^.
- Only one-third of those eligible for referral to specialized nephrology units were referred.

**Potential impact:**

- CKD management and treatment need improvement, especially with regard to blood pressure control and among individuals in the early stages of the disease.
- Greater focus on weight management and statin therapy is required for better cardiovascular disease prevention.
- Recognizing CKD is an essential critical first step toward optimizing CKD care.

## INTRODUCTION

Chronic kidney disease (CKD), with a prevalence of >10% worldwide, is a growing global health challenge and a major contributor to mortality [1]. Even in its early stages, CKD is associated with an elevated risk of cardiovascular disease (CVD), which is the leading cause of excess mortality in individuals with CKD [2]. Early recognition of CKD is therefore critical to prevent both cardiovascular complications and the progression to kidney failure. The most important progression promoters of CKD are uncontrolled hypertension and a high level of albuminuria [3]. Renin–angiotensin–system inhibitors (RASi) are particularly effective renoprotective therapies because they reduce both blood pressure (BP) and albuminuria, while simultaneously lowering CVD risk [4].

In the Danish population-based Lolland-Falster Health Study (LOFUS) [5], conducted in an area with life expectancy and socioeconomic status below the national average, we recently identified a CKD prevalence of 18% [6]. Most individuals with CKD were in stages 1–3. Strikingly, overall CKD recognition was only 7.1%, and CKD awareness was as low as 4.4% [6]. This highlights a critical gap in identifying CKD in the general population, which consequently delays appropriate initiation of renoprotective treatments, the prevention of CVD, and timely referral to specialized nephrology care. Timely referral has been proved to enhance pharmacological management, reduce hospitalizations, and lower mortality rates [7–10], yet studies show that a substantial proportion of patients starting chronic dialysis are referred late [9].

This study examines the management of individuals with CKD identified in LOFUS, with a focus on renoprotective strategies, including BP control and the use of RASi, referrals to nephrology units, and the prevention of CVD including statin treatment and lifestyle factors. This knowledge will help identify areas for improvement in CKD care and guide policy changes to enhance public health. We hypothesized that guideline-based treatment and management of CKD is suboptimal, with <50% exhibiting BP control, <50% of those eligible for RASi and statins receiving these treatments, and <50% reporting non-smoking or having a BMI <25 kg/m².

## MATERIALS AND METHODS

This cross-sectional study uses data from LOFUS, a household and population-based study conducted in Lolland-Falster, a rural-provincial area in Denmark with 103 000 inhabitants [5]. Randomly selected individuals and their household members were invited, with 36% of them choosing to participate [11]. All adult participants (*n* = 16 142) visited one of three study sites once between 2016 and 2020, where they underwent a physical examination and provided blood and urine samples. Before their visit, they completed a self-administered questionnaire. LOFUS is described in detail in the published study protocol [5]. Data were subsequently linked with Danish health registers.

### Study sample

Participants aged ≥18 years from LOFUS with CKD were included, excluding those without eGFR measurements. CKD was defined as albuminuria [urine albumin-creatinine ratio (UACR) ≥30 mg/g from spot-urine sample], an eGFR <60 ml/min/1.73 m^2^, or a registered diagnosis consistent with CKD (ICD codes listed in Table S1) in the Danish National Patient Register (DNPR) [12]. UACR and eGFR were measured at a single time point and eGFR was calculated using the 2009 creatinine-based CKD-EPI equation [13]. Individuals with acute illness could reschedule their study appointment, minimizing the likelihood that acute kidney injury influenced the measurements. Diagnostic codes were used to identify individuals with CKD and normal biochemical markers, such as those with structural abnormalities of the kidney. Recognized CKD was defined as a registered CKD diagnosis or as self-reported kidney disease in the questionnaire.

### Blood pressure measurements

BP was measured using an electronic BP device (Connex ProBP 3400, Welch Allyn, USA) during the study visit. Participants were positioned supine and rested for 5 minutes before the first measurement and were instructed to avoid moving or speaking. The last of three consecutive measurements was used for analysis.

### Comorbidities and socioeconomic status

CVD was defined as a diagnosis of ischemic heart disease, heart failure, atrial fibrillation, peripheral vascular disease, or stroke, either self-reported or recorded in the DNPR (Table S1). Diabetes was defined by HbA1c level ≥48 mmol/mol, self-reported diagnosis or use of antidiabetic medication, or a registered diabetes diagnosis [14]. Abdominal obesity was defined as waist circumference ≥88 cm for women or ≥102 cm for men [15]. Educational level and occupational status were self-reported and used as indicators of socioeconomic status. Response options for each question, along with their categorization, are listed in Table S2.

### Outcome definitions

#### Renoprotection

Renoprotective strategies were evaluated by BP levels and RASi use. BP control was defined according to the Danish national CKD guidelines [16] at the time of the study:

For individuals without diabetes:

- controlled BP was defined as <140/90 mmHg when UACR was <300 mg/g,
- or <130/80 mmHg when UACR was ≥300 mg/g.

For individuals with diabetes:

- controlled BP was defined as <140/90 mmHg when UACR was <30 mg/g,
- or <130/80 mmHg when UACR was ≥30 mg/g.

RASi treatment was defined as a redeemed prescription of angiotensin-converting enzyme inhibitors or angiotensin II receptor blockers in the Danish Register of Pharmaceutical Sales [17] within 12 months before the study visit. RASi was recommended for individuals with indication for BP-lowering drugs and UACR ≥30 mg/g and concomitant diabetes, as well as for those without diabetes and UACR >700 mg/g [16]. Notably, this threshold was more conservative than the 2012 Kidney Disease: Improving Global Outcomes (KDIGO) recommendation of initiating RASi at UACR >300 mg/g in individuals without diabetes [18].

More recently, medications with additional cardio- and renoprotective benefits—such as sodium-glucose co-transporter 2 inhibitors—have been identified and incorporated into clinical guidelines as add-on therapy to RASi [19]. Since these recommendations emerged after the LOFUS study period, use of sodium-glucose co-transporter 2 inhibitors was not defined as an outcome in this study but, is reported in Table S3.

#### Cardiovascular disease prevention

Statins were indicated for individuals aged 50 and older or for younger individuals with CVD risk factors such as diabetes, prior ischemic stroke, coronary artery disease, or an estimated 10-year risk of coronary death or non-fatal myocardial infarction greater than 10% [20]. Statin treatment was defined as a redeemed prescription within 12 months prior to the study appointment. Lifestyle factors were assessed by BMI (<25 kg/m^2^) and non-smoking. Data on smoking were self-reported and divided into two categories for analyses: non-smoking (never or previous) and current smoking (daily, once a week, or less than once a week).

#### Referral to specialized nephrology care

National guidelines recommended referral to a nephrology unit for individuals with UACR >700 mg/g or eGFR <30 ml/min/1.73 m^2^ persisting >3 months [16]. Referral was defined as outpatient contact documented in the DNPR within 10 years before the study appointment. Denmark has a tax-financed universal healthcare system with free hospital access and no private nephrology clinics.

### Statistics

Data processing and analysis were performed using R Statistical Software (version 4.3.3) [21]. Categorical variables were expressed as proportions and continuous variables as means with standard deviations or medians with interquartile ranges where appropriate. Observations with fewer than five cases were labeled as “too few to specify” per Danish Health Data Authority's guidelines. Generalized linear mixed-effects models with household as a random effect were used to estimate the association between CKD stages and outcomes, providing odds ratios (ORs) and 95% confidence intervals (CIs). These models accounted for clustering, as 70% of individuals in the study participated with one or more of their household members [6]. Standard logistic regression models were used to analyze individuals with an indication of nephrology referral, as household clustering in this group was limited. Individuals with missing data were excluded in the analyses. Multivariable models included age (<65 vs. ≥65 years), sex, socioeconomic status, and diabetes as explanatory variables.

### Ethics

All participants provided written informed consent. LOFUS has been approved by the Region of Zealand's Ethical Committee on Health Research (SJ-421) and is registered with ClinicalTrials.gov (NCT02482896). Both LOFUS and the current study are registered with the Danish Data Protection Agency (nos. P-2024-16360 and P-788-2022).

## RESULTS

Of the 2903 individuals identified with CKD in LOFUS, 22 were excluded due to missing eGFR data. The current study therefore included 2881 individuals, of whom 57.6% were women. Median age was 67.8 years. Demographic and clinical characteristics of the study participants are presented in Table 1, stratified by CKD stages. Most individuals had CKD stage 1 or 2 (32.1% and 39.2%, respectively), 21.5% had stage 3a, while <8% had stages 3b, 4, or 5. Only a few had previously undergone kidney transplantation (<1%), and none received treatment with chronic dialysis. Albuminuria testing was performed in 98.4%, and recognized CKD was identified in 5% of individuals in stages 1–2, 6.6% in stage 3a, and 29.5% in stages 3b–5. Regarding comorbidities, 14.4% had diabetes and 57.4% had abdominal obesity. Individuals with diabetes had a higher prevalence of CVD and albuminuria compared to those without diabetes (Table S3). Overall, one-fifth of the participants had CVD, with its prevalence increasing from 10% in stage 1, to 20% in stage 2, 31% in stage 3b, and 47% in stages 3–5. Ischemic heart disease was the most common CVD diagnosis, accounting for >60% of the cases. The distribution of other CVD diagnoses, such as heart failure and atrial fibrillation, is detailed in Table 1.

**Table 1:** Demographic and clinical characteristics of the participants.

*Characteristics*	Total	Stage 1: eGFR ≥90 ml/min/1.73m^2^	Stage 2: eGFR 60–89 ml/min/1.73 m^2^	Stages 3a: eGFR 45–59 ml/min/1.73 m^2^	Stages 3b–5: eGFR <45 ml/min/1.73 m^2^
*N*	2 881	925	1 130	619	207
Female sex, *n* (%)	1 659 (57.6)	599 (64.8)	605 (53.5)	353 (57.0)	102 (49.3)
Age (years), median (IQR)	67.8 (56.7–75.1)	54.1 (42.5–63.3)	70.7 (63.4–76.4)	73.1 (67.3–78.9)	77.3 (71.3–83.1)
Age distribution, *n* (%)					
18–55	693 (24.1)	508 (54.9)	139 (12.3)	32 (5.2)	14 (6.8)
56–65	607 (21.1)	252 (27.2)	237 (21.0)	102 (16.5)	16 (7.7)
66–75	929 (32.2)	149 (16.1)	459 (40.6)	257 (41.5)	64 (30.9)
75+	652 (22.6)	16 (1.7)	295 (26.1)	228 (36.8)	113 (54.6)
Postsecondary education, *n* (%)					
Medium/long	622 (23.0)	224 (26.1)	263 (24.5)	108 (18.7)	27 (14.3)
Short	1 491 (55.2)	456 (53.2)	597 (55.6)	144 (24.9)	51 (27.0)
No	586 (21.7)	177 (20.7)	214 (19.9)	327 (56.5)	111 (58.7)
Occupational status, *n* (%)					
Active	955 (34.9)	527 (61.1)	300 (27.6)	128 (16.3)	
Temporarily not active	70 (2.6)	Too few to specify			
Not active	1 652 (60.4)	256 (29.7)	760 (69.9)	636 (80.8)	
Other	60 (2.2)	30 (3.5)	12 (1.1)	18 (2.3)	
CVD, *n* (%)	605 (21.0)	92 (9.9)	224 (19.8)	192 (31.0)	97 (46.9)
Atrial fibrillation, *n* (%)	191 (6.6)	17 (1.8)	76 (6.7)	68 (11.0)	30 (14.5)
Stroke, *n* (%)	57 (2.0)	5 (0.5)	24 (2.1)	20 (3.2)	8 (3.9)
Heart failure, *n* (%)	77 (2.7)	10 (1.1)	21 (1.9)	24 (3.9)	22 (10.6)
Peripheral vascular disease, *n* (%)	100 (3.4)	13 (1.4)	38 (3.4)	31 (5.0)	17 (8.2)
Ischemic heart disease, *n* (%)	377 (13.1)	67 (7.2)	127 (11.2)	118 (19.1)	65 (31.4)
Diabetes, *n* (%)	414 (14.4)	125 (13.5)	145 (12.8)	93 (15.0)	51 (24.6)
Abdominal obesity^a^, *n* (%)	1 653 (57.4)	472 (51.0)	627 (55.5)	399 (64.5)	155 (74.9)
BMI category, *n* (%)					
<25 kg/m^2^	919 (32.2)	353 (38.6)	389 (34.8)	143 (23.3)	34 (16.6)
25.0–29.9 kg/m^2^	1 075 (37.7)	286 (31.3)	427 (38.2)	281 (45.8)	81 (39.5)
≥30.0 kg/m^2^	857 (30.1)	276 (30.2)	302 (27.0)	189 (30.8)	90 (43.9)
Systolic BP (mmHg), mean (SD)	140 (21.8)	137 (22.5)	144 (21.5)	138 (20.2)	138 (21.2)
Diastolic BP (mmHg), mean (SD)	80.6 (10.0)	81.9 (10.7)	81.3 (9.72)	78.2 (8.72)	77.5 (10.1)
BP ≥130/80 mmHg, *n* (%)	2 045 (71.5)	607 (65.8)	881 (78.5)	419 (68.5)	138 (67.3)
BP ≥140/90 mmHg, *n* (%)	1 386 (48.1)	397 (43.1)	623 (55.5)	274 (44.3)	92 (44.4)
CKD stages, *n* (%)					
1: eGFR ≥90 ml/min/1.73 m^2^	925 (32.1)				
2: eGFR 60–89 ml/min/1.73 m^2^	1 130 (39.2)				
3a: eGFR 45–59 ml/min/1.73 m^2^	619 (21.5)				
3b: eGFR 30–44 ml/min/1.73 m^2^	173 (6.0)				
4–5: eGFR <30 ml/min/1.73 m^2^	34 (1.2)				
UACR category, *n* (%)					
<30 mg/g	541 (19.1)	21 (2.3)	19 (1.7)	414 (70.1)	87 (45.1)
30 to <300 mg/g	2 103 (74.2)	836 (90.5)	1 042 (92.4)	156 (26.4)	69 (35.8)
300 to <700 mg/g	120 (4.2)	48 (5.2)	45 (4.0)	11 (1.9)	16 (8.3)
≥700 mg/g	72 (2.5)	19 (2.1)	22 (2.0)	10 (1.7)	21 (10.9)
Recognized CKD, *n* (%)	204 (7.1)	45 (4.9)	57 (5.0)	41 (6.6)	61 (29.5)
CKD diagnosis identified by, *n* (%)					
Biochemical data (UACR/eGFR)	2838 (98.5)	903 (97.6)	1109 (98.1)	619 (100)	207 (100)
Registered diagnosis only^b^	43 (1.5)	22 (2.4)	21 (1.9)	0 (0)	0 (0)
Nephrology unit referral indicated, *n* (%)	99 (3.4)	19 (2.1)	22 (1.9)	10 (1.6)	48 (23.2)
Receiving RASi, *n* (%)	1 013 (35.2)	188 (20.3)	396 (35.0)	292 (47.2)	137 (66.2)
RASi indicated, *n* (%)	367 (12.7)	126 (13.6)	145 (12.8)	48 (7.8)	48 (23.2)
Receiving statins, *n* (%)	793 (27.5)	151 (16.3)	341 (30.2)	209 (33.8)	92 (44.4)
Statins indicated, *n* (%)	2 459 (85.4)	589 (63.7)	1 057 (93.5)	813 (98.4)	
Smoking, *n* (%)					
Never	1 108 (41.1)	360 (42.6)	432 (40.2)	246 (41.9)	70 (37.0)
Former	1 085 (40.3)	235 (27.8)	473 (44.0)	276 (47.0)	101 (53.4)
Current	502 (18.6)	250 (29.6)	169 (15.7)	65 (11.1)	18 (9.5)

a Waist circumference ≥88 cm for women and ≥102 cm for men.

b Among the 43 individuals with normal eGFR and UACR, 23 had cystic kidney disease (Q61), 9 had other disorders of kidney and ureter, not elsewhere classified (N28), and the remaining (too few to specify) had diagnoses such as E10.2, N03, N05, N06, N18, N27, and Q62.

A higher proportion of individuals had medium or long postsecondary education in stage 1 (26.1%) and stage 2 (24.5%), compared to those in stages 3–5 (17.6%). Additionally, individuals with an inactive occupational status represented <30% of those in stage 1, and >80% of those in stages 3 to 5, where the median age was highest.

### Outcomes

Details of the various study outcomes are presented in Table 2. Less than half (47.5%) of individuals exhibited BP control according to the defined targets. This proportion was 51.2% in stage 3a and decreased slightly to 45.9% in stages 3b–5. Accordingly, the likelihood of controlled BP was significantly higher in stage 3a (OR, 1.81; 95% CI, 1.35–2.44) and in stages 3b–5 (OR, 1.68; 95% CI, 1.12–2.52), compared to stage 1. Comparable proportions of individuals with controlled BP were observed in recognized and unrecognized CKD (43% vs. 48%), however, controlled BP was twice as common in individuals without diabetes than in those with diabetes (Table S3). Overall, 35% of participants received RASi and among the 367 individuals with an indication for RASi therapy, 72.8% were treated. Multivariable analyses showed a higher likelihood of receiving RASi when indicated in stages 3b–5 (OR, 7.91; 95% CI, 2.14–29.18), compared to stage 1.

**Table 2:** Outcome variables.

	Total	Stage 1: eGFR ≥90 ml/min/1.73 m^2^	Stage 2: eGFR 60–89 ml/min/1.73 m^2^	Stages 3a: eGFR 45–60 ml/min/1.73 m^2^	Stages 3b–5: eGFR <45 ml/min/1.73 m^2^
BP control, *n* (%)	1 369 (47.5)	487 (52.6)	470 (41.6)	317 (51.2)	95 (45.9)
Receiving RASi when indicated, *n* (%)	267 (72.8)	83 (65.9)	105 (72.4)	37 (77.1)	42 (87.5)
Receiving statins when indicated, *n* (%)	791 (32.2)	150 (25.5)	340 (32.2)	209 (34.3)	92 (45.1)
Non-smoking, *n* (%)	2 193 (81.4)	595 (70.4)	905 (84.3)	554 (88.9)	189 (90.5)
BMI <25 kg/m^2^, *n* (%)	919 (32.2)	353 (38.6)	389 (34.8)	143 (23.3)	34 (16.6)
Referral to a nephrology unit when indicated, *n* (%)	33 (33.3)	Too few to specify			

Statin therapy was indicated in 2459 individuals, of whom only a third received treatment, and multivariable analyses indicated significantly higher statin use in stages 3a and 3b–5 compared to stage 1. Treatment with statins and RASi were more common in individuals with coexisting diabetes (68% and 74%) than in those without (25% and 60%) (Table S3).

Regarding lifestyle factors, less than one-third of the participants had a BMI below 25 kg/m^2^ and in stages 3–5, the likelihood of BMI below 25 kg/m^2^ was significantly lower than in stage 1. In contrast, over 80% self-reported non-smoking, rising to >90% in stages 3b–5. The proportion of former smokers also increased across stages.

Of all those who met the criteria for nephrology referral (*n *= 99), one-third had been in contact with a nephrology unit. OR of being referred to a nephrologist was significantly higher in stages 3b–5 than in stage 1 (Table 3).

**Table 3:** Analyses of the association between CKD stages and the different outcomes.

	Stage 1: eGFR ≥90 ml/min/1.73 m^2^	Stage 2: eGFR 60–89 ml/min/1.73 m^2^	Stages 3a: eGFR 45–59 ml/min/1.73 m^2^	Stages 3b–5: eGFR <45 ml/min/1.73 m^2^
Model^a^	Reference	OR (95% CI)	OR (95% CI)	OR (95% CI)
BP control				
1. Unadjusted model	Reference	0.60 (0.48–0.74)***	0.94 (0.74–1.19)	0.73 (0.51–1.04)
2. Sex and age	Reference	0.95 (0.75–1.19)	1.63 (1.23–2.16)**	1.39 (0.95–2.04)
3. Socioeconomic parameters	Reference	0.94 (0.75–1.19)	1.82 (1.35–2.44)***	1.52 (1.02–2.27)*
4. Diabetes	Reference	0.91 (0.72–1.16)	1.81 (1.35–2.44)***	1.68 (1.12–2.52)*
Receiving RASi when indicated				
1. Unadjusted model	Reference	1.37 (0.80–2.34)	1.76 (0.80–3.87)	3.71 (1.42–9.72)**
2. Sex and age	Reference	1.29 (0.73–2.29)	1.69 (0.75–3.82)	3.61 (1.36–9.58)*
3. Socioeconomic parameters	Reference	1.32 (0.72–2.43)	2.00 (0.81–4.95)	5.23 (1.55–17.65)**
4. Diabetes	Reference	1.47 (0.79–2.72)	2.40 (0.95–6.07)	7.91 (2.14–29.18)**
Receiving statins when indicated				
1. Unadjusted model	Reference	1.43 (1.11–1.86)**	1.62 (1.21–2.17)***	2.68 (1.79–4.03)***
2. Sex and age	Reference	1.05 (0.80–1.39)	1.17 (0.86–1.60)	1.80 (1.19–2.72)**
3. Socioeconomic parameters	Reference	1.04 (0.77–1.41)	1.15 (0.82–1.61)	1.73 (1.10–2.72)*
4. Diabetes	Reference	1.30 (0.92–1.84)	1.36 (0.92–2.01)	1.77 (1.06–2.96)*
Non-smoking				
1. Unadjusted model	Reference	5.50 (3.34–9.08)***	13.05 (6.81–24.98)***	16.98 (6.00–48.07)***
2. Sex and age	Reference	5.76 (3.18–10.46)***	13.21 (6.33–27.57)***	16.51 (5.44–50.08)***
3. Socioeconomic parameters	Reference	6.07 (3.31–11.12)***	14.25 (6.63–30.65)***	17.90 (5.80–55.28)***
4. Diabetes	Reference	5.33 (3.00–9.49)***	12.30 (5.83–25.92)***	16.33 (5.36–49.75)***
BMI <25 kg/m^2^				
1. Unadjusted model	Reference	0.85 (0.71–1.02)	0.48 (0.38–0.61)***	0.31 (0.21–0.47)***
2. Sex and age	Reference	0.89 (0.72–1.09)	0.48 (0.37–0.62)***	0.33 (0.22–0.50)***
3. Socioeconomic parameters	Reference	0.90 (0.73–1.12)	0.51 (0.39–0.67)***	0.33 (0.21–0.52)***
4. Diabetes	Reference	0.87 (0.70–1.09)	0.50 (0.38–0.65)***	0.35 (0.22–0.54)***
Referral to a nephrology unit when indicated				
1. Unadjusted model	Reference	0.85 (0.11–6.69)	5.67 (0.82–39.27)	9.24 (1.92–44.44)**
2. Sex and age	Reference	1.21 (0.15–10.07)	7.79 (1.04–58.61)*	15.02 (2.72–83.02)**
3. Socioeconomic parameters	Reference	2.26 (0.13–39.56)	19.83 (0.98–401.59)	36.83 (2.38–568.68)**
4. Diabetes	Reference	2.32 (0.13–41.94)	20.22 (0.98–418.87)	37.70 (2.36–602.50)**

***P ≤ .001, **P ≤ .01, *P ≤ .05.

a Model 1, unadjusted. Model 2, adjusted for age and sex (reference male sex). Model 3, further adjusted for occupational status and educational level. Model 4, further adjusted for diabetes.

## DISCUSSION

In this study of 2881 individuals with CKD from the population-based LOFUS, we identified suboptimal adherence to guideline-based CKD management, particularly in relation to BP control, CVD prevention, and nephrology referrals. However, it appeared to be significantly more optimal in CKD stages 3b–5 compared to the earlier stages and in individuals with recognized CKD compared to those with unrecognized CKD. Contrary to our hypothesis, the use of RASi when indicated was high at >70%.

BP control is a cornerstone in management of CKD, as it slows down progression of kidney damage and reduces CVD risk. However, our results confirm the well-documented challenge of uncontrolled BP in individuals with CKD [22–24], with <50% having BP below targets. The likelihood of BP control was higher in stages 3–5 compared to stage 1, possibly due to greater disease recognition in later stages. Our previous study on the LOFUS cohort showed that disease awareness in hypertension exceeded 50%, while awareness in CKD was below 5% [6]. When these conditions coexist, recognizing both is essential, as BP targets vary depending on the level of albuminuria [25].

The suboptimal BP control observed in LOFUS support findings from a Canadian study that reported an association between living in rural areas and failure to meet BP targets [26] possibly explained by reduced access to healthcare, socioeconomic disparities, or differences in provider practices. However, the criteria for defining geographical areas, such as what qualifies as “rural,” can vary between countries and affect comparability.

Importantly, LOFUS was conducted prior to the Systolic Blood Pressure Intervention Trial, whose findings has led to recommendations of intensive BP lowering in the 2024 KDIGO guidelines (systolic BP <120 mmHg), as it demonstrates benefits in terms of CVD and mortality in individuals with CKD [19]. The new BP recommendations will further pose a challenge with BP control in individuals with CKD.

Although not always recognized as such in real-world practice, individuals with CKD should be regarded as one of the highest-risk groups for CVD [27]. Accordingly, in our study, CVD was present in almost half of individuals in stages 3b–5, and even at stage 2, one in five had CVD. These numbers are likely underestimated, as CVD often goes undiagnosed in patients with CKD, partly due to the atypical presentation of ischemic heart disease in this population, where many patients are asymptomatic, despite major acute ischemic events [27, 28]. Our findings underscore the dual importance of early CKD recognition and management of cardiovascular risk factors in individuals with CKD.

Treatment with statins and RASi, individually reduce mortality and cardiovascular events in patients with CKD, particularly in the early stages [4, 29]. Additionally, RASi lower the risk of kidney failure by managing hypertension and proteinuria. Similar to observations in two recent Danish cohort studies [30, 31], and contrary to our hypothesis, a substantial proportion of individuals with indication for RASi (72.8%) received treatment in this study.

International studies [26, 32, 33] have consistently reported lower RASi use in CKD cohorts; however, these estimates typically reflect overall use, without considering whether treatment was clinically indicated. In contrast, our study, assessed RASi use in relation to guideline recommendations, providing a more meaningful measure of treatment quality. The higher treatment rates observed in our study may also reflect the more conservative threshold for initiating RASi treatment in the Danish guidelines compared to KDIGO.

In line with two large multinational studies [34, 35], we found that RASi treatment was more common in individuals with concomitant diabetes than those without. Similarly, studies consistently report higher levels of albuminuria testing in patients with diabetes than those without [33, 36]. In a recent study by Chu *et al*., albuminuria testing was associated with higher OR of receiving RASi (OR, 2.39; 95% CI, 2.32–2.46) [36]. Thus, the lower frequency of RASi treatment in CKD without diabetes may partly be explained by a lack of testing for albuminuria prior to our study, resulting in a proportion with undiagnosed albuminuria where treatment would be indicated. Additionally, higher albuminuria and CVD prevalence in individuals with diabetes may also contribute to the observed treatment differences.

In our study, only one-third of individuals received adequate statin treatment, likely reflecting the high proportion of unrecognized CKD. Even in the Copenhagen CKD cohort (>700 patients recruited from a nephrology unit), where CKD was fully recognized, just over half received statins, with some undertreatment linked to discontinuation due to side effects, while most, no clear reason for non-treatment was identified [23].

Overweight or obesity was present in two-thirds of the individuals in our study, aligning with findings from previous European studies [22, 23, 37]. This is noteworthy, as obesity is usually more prevalent in later CKD stages [23], and <30% were in stages 3–5 in our cohort compared to >70% in the other European studies. Targeted CKD screening in individuals with overweight/obesity during visits to general practitioners, particularly in populations like ours with a high prevalence of CKD and obesity, could be a feasible strategy for early intervention.

Encouragingly, <20% of participants smoked and those with more advanced CKD stages were more likely to be former smokers. This trend may be attributed to participation bias [38] or to a higher proportion of individuals with recognized CKD in later stages, who might have quit smoking following diagnosis and healthcare advice. Counseling on smoking cessation and weight loss is an essential component in CKD care, as both are modifiable risk factors for CKD and CVD.

Referral to nephrology units was limited in our study, with only a third of eligible individuals seen by specialists. However, the absence of a confirmatory UACR or eGFR measurement after >3 months and the small sample size (*n *= 99) warrants caution when interpreting these results. Another Danish study by Kampmann *et al*., found that only 16% of individuals with CKD stage 4–5 were referred [39]. The authors noted that individuals residing in communities with a nephrology department or those with higher education were more likely to receive specialized CKD care [39]. In our study, <20% of individuals in stages 3–5 had a medium or long postsecondary education. Furthermore, the region that includes Lolland-Falster, has the lowest number of specialist doctors, including general practitioners, per 1000 inhabitants in the country, which likely affects access to care [40]. Factors among physicians that may influence referral patterns include workload constraints, decisions to withhold referrals due to multimorbidity or limited life expectancy, or the lack of testing for eGFR or UACR resulting in unknown CKD severity [22].

It is, however, important to acknowledge the growing burden on general practitioners as the CKD population expands, new treatment options emerge, and guidelines become increasingly complex. Consequently, policy interventions should focus on supporting and empowering general practitioners, particularly in non-urban areas with high disease burdens and limited healthcare resources.

### Strengths and limitations

Our study has certain limitations. First, due to LOFUS's cross-sectional design, only single measurements of eGFR and UACR were available, whereas the KDIGO guidelines recommend two abnormal values at least 3 months apart for diagnosis [19]. Consequently, we cannot fully exclude that a proportion of individuals may have had transient changes due to acute illness. However, unlike register-based studies, our measurements were collected as a part of a population-based study, where participants were not selected based on clinical indication. Moreover, individuals who were acutely ill had the opportunity to reschedule their study visit, making it unlikely that acute illness substantially biased our findings. The same applies to BP, measured on a single occasion (although three consecutive measurements), carrying a risk of false positive results. Second, while invited participants in LOFUS were randomly selected, the 36% participation proportion introduces selection bias, an inherent limitation of health examination studies. For example, women were more likely to participate than men (38% vs. 33%), with the highest participation among those aged 60–69 years and the lowest among those aged 90 years and older [11]. Non-participants were also more likely to be publicly supported and had a threefold higher mortality rate than that of participants [38]. Therefore, our findings likely underestimate the true gap in adherence to CKD guidelines in Lolland-Falster.

Despite these limitations, our study has notable strengths. To our knowledge, it is the first in Denmark to examine the treatment and management of CKD in a non-urban area and using data from a comprehensive health examination survey, rather than relying solely on register data. The inclusion of a large proportion of individuals with unrecognized early-stage CKD, provides a more accurate representation of CKD within the broader population than most CKD cohorts, which primarily include individuals in later stages.

## CONCLUSION

Our findings reveal a gap between guideline-recommended CKD management and actual treatment practices in Lolland-Falster, Denmark. Suboptimal BP control and statin use, unmet BMI targets, and limited nephrology referrals highlight opportunities to improve management and treatment of CKD, particularly in early stages. Encouragingly, RASi use was high among those with an indication, which may reflect increasing awareness of its benefits in clinical practice. Nevertheless, >90% of individuals with CKD in this cohort were unrecognized, underscoring the need for early recognition as a critical first step toward optimizing CKD care.

## Supplementary Material

sfaf242_Supplemental_File

## Data Availability

Data from third parties are not publicly available but can be requested from LOFUS, Statistics Denmark and the Danish Health Data Authority, in compliance with Danish legislation.
