# Correction to: Management and treatment of chronic kidney disease in the Danish Lolland-Falster Health Study: focus on renoprotection and cardiovascular disease prevention

**DOI:** 10.1093/ckj/sfag135

**Published:** 2026-05-11

This is a correction to: Ebba Mannheimer, Morten Buus Jørgensen, Kristine Hommel, Anne-Lise Kamper, Randi Jepsen, Bo Feldt-Rasmussen, Mads Hornum, Management and treatment of chronic kidney disease in the Danish Lolland-Falster Health Study: focus on renoprotection and cardiovascular disease prevention, *Clinical Kidney Journal*, Volume 18, Issue 9, September 2025, sfaf242, https://doi.org/10.1093/ckj/sfaf242

In the originally published manuscript, the age distribution labels in Table 1 were erroneously given as: 18–45, 46–65, 66–75, 75+.

These have been corrected to read: 18-55, 56-65, 66-75, 75+.

