# Supplementary material for: Management and treatment of chronic kidney disease in the Danish Lolland-Falster Health Study: focus on renoprotection and cardiovascular disease prevention
Source: Clin Kidney J. 2025 Aug 1;18(9):sfaf242. doi: 10.1093/ckj/sfaf242 (PMC12399973; doi:10.1093/ckj/sfaf242)
Supplement: sfaf242_Supplemental_File [file sfaf242_supplemental_file.pdf]

Supplementary

Table S1. ICD-10 codes and respective diagnoses used to identify individuals with CKD and other comorbidities

Chronic kidney disease

BJFD2	Dialysis in chronic kidney disease
E10.2	Type 1 diabetes mellitus with kidney complications
E11.2	Type 2 diabetes mellitus with kidney complications
E12.2	Malnutrition-related diabetes mellitus with kidney complications
E13.2	Other specified diabetes mellitus with kidney complications
E14.2	Unspecified diabetes mellitus with kidney complications
G62	Congenital obstructive defects of renal pelvis and congenital malformations of ureter
I12	Hypertensive chronic kidney disease
I13	Hypertensive heart and chronic kidney disease
N00	Acute nephritic syndrome
N01	Rapidly progressive nephritic syndrome
N02	Recurrent and persistent hematuria
N03	Chronic nephritic syndrome
N04	Nephrotic syndrome
N05	Unspecified nephritic syndrome
N06	Isolated proteinuria with specified morphological lesion
N07	Hereditary nephropathy, not elsewhere classified
N08.3	Glomerular disorders in diabetes mellitus
N08.4	Glomerular disorders in other endocrine, nutritional, and metabolic diseases
N08.5	Glomerular disorders in systemic connective tissue disorders
N11	Chronic tubulo-interstitial nephritis
N11.0	Nonobstructive reflux-associated chronic pyelonephritis
N11.8	Other chronic tubulo-interstitial kidney disease
N12	Tubulo-interstitial nephritis
N15.0	Balkan nephropathy
N16.2	Renal tubulo-interstitial disorders in blood diseases and disorders involving the immune mechanism
N16.3	Renal tubulo-interstitial disorders in metabolic diseases
N16.4	Renal tubulo-interstitial disorders in systemic connective tissue disorders
N18 (1–5+9)	Chronic Kidney Disease
N19	Unspecified kidney failure
N26	Unspecified contracted kidney
N27	Small kidney of unknown cause
N28	Other disorders of kidney and ureter, not elsewhere classified
N29	Other disorders of kidney and ureter in diseases classified elsewhere
Q60	Renal agenesis and other reduction defects of kidney
Q61	Cystic kidney disease
Q62	Congenital obstructive defects of renal pelvis and congenital malformations of ureter
Q63	Other congenital malformations of kidney
Q64	Other congenital malformations of urinary system
Z94.0	Kidney transplant status
Z99.2	Dependence on renal dialysis

Hypertensive diseases

I10	Essential (primary) hypertension
I11	Hypertensive heart disease
I12	Hypertensive renal disease
I13	Hypertensive heart and renal disease
I15	Secondary hypertension

Stroke

I60	Subarachnoid hemorrhage
I61	Intracerebral hemorrhage
I62	Other nontraumatic intracranial hemorrhage
I63	Cerebral infarction
I64	Stroke, not specified as hemorrhage or infarction

Ischemic heart disease

I20	Angina pectoris
I21	Acute myocardial infarction
I22	Subsequent myocardial infarction
I23	Certain current complications following acute myocardial infarction
I24	Other acute ischemic heart diseases
I25	Chronic ischemic heart disease

Heart failure

I11.0	Hypertensive heart disease with (congestive) heart failure
I13.0	Hypertensive heart and renal disease with (congestive) heart failure
I13.2	Hypertensive heart and renal disease with both (congestive) heart failure and renal failure
I42.0	Dilated cardiomyopathy
I42.6	Alcoholic cardiomyopathy
I42.7	Cardiomyopathy due to drugs and other external agents
I42.8	Other cardiomyopathies
I42.9	Cardiomyopathy, unspecified
I50	Heart failure
I50.0	Congestive heart failure
I50.1	Left ventricular failure
I50.9	Heart failure, unspecified

Other forms of heart disease

I48	Atrial fibrillation and flutter
I44	Atrioventricular and left bundle-branch block

Peripheral vascular disease

I17.0	Atherosclerosis
I17.1	Aortic aneurysm and dissection
I17.2	Other aneurysm and dissection
I17.3	Other peripheral vascular diseases
I17.4	Arterial embolism and thrombosis
I17.7	Other disorders of arteries and arterioles

Diabetes

E10	Type 1 diabetes mellitus
E11	Type 2 diabetes mellitus
E12	Malnutrition-related diabetes mellitus
E13	Other specified diabetes mellitus
E14	Unspecified diabetes mellitus

**Table S2. Categorization of socioeconomic data**
Data on socioeconomic status were obtained from the questionnaire. The question regarding occupational status had 16 different response options. However, these were divided into the following four categories in the analyses:
• ***Active:*** employee; self-employed; combined employee and self-employed; in the military; secondary school pupil; postsecondary student; apprentice; assisting spouse; housewife/househusband
• ***Temporarily Inactive:*** unemployed; undergoing rehabilitation; on sickness leave for 3 months or more
• ***Inactive****:* retired due to age; recipient of disability benefit; early retirement
• ***Other****:* other
Seven different response options for educational level were divided into the following three categories for analyses:
• ***No Postsecondary Education***
• ***Short Postsecondary Education****:* unspecified other education; one or more short courses; vocational education; short higher education for 2–3 years
• ***Medium or Long Postsecondary Education***: medium higher education for 3–4 years; long higher education for > 4 years

Table S3. Clinical characteristics of the participants stratified by diabetes status and recognized/unrecognized CKD

	Diabetes	No diabetes	Recognized CKD	Unrecognized CKD
**N**	414	2,467	204	2,677
**UACR category - n (%)**				
**<30 mg/g**	62 (15.3)	479 (19.7)	66 (33.7)	475 (18.0)
**30 to <300 mg/g**	274 (67.5)	1,829 (75.3)	87 (44.4)	2,016 (76.4)
**300 to <700 mg/g**	41 (10.1)	79 (3.3)	19 (9.7)	101 (3.8)
**<700 mg/g**	29 (7.1)	43 (1.8)	24 (12.2)	48 (1.8)
**Cardiovascular disease – n (%)**	139 (33.6)	466 (18.9)	62 (30.4)	543 (20.3)
**Blood pressure control – n (%)**	99 (23.9)	1270 (51.5)	88 (43.1)	1281 (47.9)
**Receiving RASi – n (%)**	308 (74.4)	705 (28.6)	118 (57.8)	895 (33.4)
**Receiving RASi when indicated – n (%)**	245 (74.2)	22 (59.5)	57 (91.9)	210 (68.9)
**Receiving statins – n (%)**	281 (67.9)	512 (20.8)	78 (38.2)	715 (26.7)
**Receiving statins when indicated – n (%)**	281 (67.9)	510 (24.9)	78 (43.3)	713 (31.3)
**Non–smoking – n (%)**	302 (79.7)	1,891 (81.6)	158 (80.2)	2,035 (81.5)
**BMI <25 kg/m^2 – n (%)**	46 (11.2)	873 (35.7)	44 (21.9)	875 (33.0)
**Referral to a nephrology unit when indicated – n (%)**	12 (31.6)	21 (34.4)	Too few to specify	
**Receiving SGLT2i – n (%)**	29 (7.0)	0 (0)	Too few to specify	
**CKD, chronic kidney disease; UACR, urine albumin-creatinine-ratio; RASi, renin-angiotensin-system inhibitors; SGLT2i, sodium-glucose transport protein 2 inhibitors**				

**Table S4. Referral to nephrology unit with eGFR threshold <45 mL/min/1.73 m² as referral indication**				
	**Stage 1: eGFR ≥ 90 ml/min/1.73m²**	**Stage 2: eGFR 60–89 ml/min/1.73m²**	**Stages 3a: eGFR 45–59 ml/min/1.73m²**	**Stages 3b–5: eGFR <45 ml/min/1.73m²**
**Referral to a nephrology unit when indicated – n (%)**	48 (18.6)	Too few to specify		40 (19.3)
**Model[a]**	**Reference**	**OR (95% CI)**	**OR (95% CI)**	**OR (95% CI)**
**Referral to a nephrology unit when indicated**				
**1. Unadjusted model**	Reference	0.85 (0.11–6.69)	5.67 (0.82–39.27)	2.04 (0.45–9.17)
**2. Sex and age**	Reference	1.46 (0.18–11.94)	8.14 (1.09–60.56) *	5.05 (0.99–25.67)
**3. Socioeconomic parameters**	Reference	2.53 (0.19–34.40)	17.65 (1.30–239.53) *	9.54 (0.98–92.85)
**4. Diabetes**	Reference	2.75 (0.20–37.76)	17.86 (1.31–242.80) *	10.08 (1.02–99.41) *

***** $p \leq 0.001$, ** $p \leq 0.01$, * $p \leq 0.05$**
[a]Model 1: unadjusted. Model 2: adjusted for age and sex (reference male sex). Model 3: further adjusted for occupational status and educational level. Model 4: further adjusted for diabetes.
OR, odds ratio; CI, confidence interval
